# Integrating computer vision to prosthetic hand control with sEMG: Preliminary results in grasp classification

**DOI:** 10.3389/frobt.2022.948238

**Published:** 2022-09-23

**Authors:** Shuo Wang, Jingjing Zheng, Ziwei Huang, Xiaoqin Zhang, Vinicius Prado da Fonseca, Bin Zheng, Xianta Jiang

**Affiliations:** ^1^ Department of Computer Science, Memorial University of Newfoundland, St. John’s, NL, Canada; ^2^ Wenzhou University, College of Computer Science and Artificial Intelligence, Zhejiang, China; ^3^ University of Alberta, Department of Surgery, Edmonton, AB, Canada

**Keywords:** myoelectric prosthesis, computer vision, sEMG, grasp classification, machine learning

## Abstract

The myoelectric prosthesis is a promising tool to restore the hand abilities of amputees, but the classification accuracy of surface electromyography (sEMG) is not high enough for real-time application. Researchers proposed integrating sEMG signals with another feature that is not affected by amputation. The strong coordination between vision and hand manipulation makes us consider including visual information in prosthetic hand control. In this study, we identified a sweet period during the early reaching phase in which the vision data could yield a higher accuracy in classifying the grasp patterns. Moreover, the visual classification results from the sweet period could be naturally integrated with sEMG data collected during the grasp phase. After the integration, the accuracy of grasp classification increased from 85.5% (only sEMG) to 90.06% (integrated). Knowledge gained from this study encourages us to further explore the methods for incorporating computer vision into myoelectric data to enhance the movement control of prosthetic hands.

## 1 Introduction

Hands are one of the essential tools for humans to achieve a wide variety of manipulations. The loss of hands can be devastating to a person, depriving them of their ability to study, work, or even live a daily life (Niedernhuber et al., 2018). Amputation can lead to career shifts or even unemployment, possibly leading to more severe problems such as social isolation (Burger and Marinček, 2007; Jang et al., 2011).

To restore the fundamental abilities and the level of independence of amputees, it is a feasible option to wear non-invasive upper-limb prostheses. Compared to cosmetic and body-powered hands, myoelectric prostheses can achieve a more realistic simulation of the grasping process, providing a better user experience (Maat et al., 2018; Castellini and Smagt, 2009). In current days, an advanced myoelectric prosthesis is actuated by a classifier, which converts muscular signals into corresponding grasp gestures. The muscular signals are collected by the surface electromyography (sEMG) sensors placed on the upper limb. On one hand, the grasp classification by sEMG is well studied, and satisfied recognition accuracy has been produced by several studies (Chen et al., 2007a; Chen et al., 2007b; Jiang et al., 2017). On the other hand, the satisfactory accuracy was limited to the laboratory environment, and most research on myoelectric prostheses did not provide enough technical support for effective application improvement in the clinical and real-life environment (Farina et al., 2014; Roche et al., 2014; Resnik et al., 2018; Simon et al., 2019). The biggest obstacle of myoelectric prosthetic hands is that sEMG signals are hard to be decoded to an applicable level. In addition, its performance is also affected by other factors, such as muscle flexibility, muscle fatigue, and sweat. Therefore, the classification performance is hard to be further improved by only relying on sEMG.

In humans, vision is critical in performing hand gestures before the activity and guiding the activity itself. Humans use visual information to understand and predict coming actions (Johansson et al., 2001). Moreover, in the study by Hebert et al. (2019), it has been found that the visual interaction of amputees is more active than intact subjects. The strong relationship between vision and action makes integrating vision and muscle signals a promising prospect.

Some researchers have integrated visual information with myoelectric prostheses to improve their performance (Hao et al., 2013; Markovic et al., 2014; Markovic et al., 2015; Ghazaei et al., 2017). In these experiments, the subjects often wore an eye-tracking device, which can also record the first-person video using the integrated camera. The main idea behind these studies is to identify objects to be grasped in the video and then select the corresponding grasp gestures. However, the subjects were asked to stare at the object (Bouwsema, 2014; Sobuh et al., 2014; Hebert et al., 2019), or manually take a photo (Ghazaei et al., 2017), until it was recognized and then grasp it. In these cases, the visual information is obtained by established rules that the subject must follow, such as staring at the object for at least 3 s, which is not a natural way to perform the grasp action.

This study investigates how the grasp classification accuracy changes over the entire grasping process while identifying a period that can achieve the best grasp classification outcome using visual data. We call this interval the *sweet period*. The sweet period should also be short and located in the early phases in order to speed up the control process. Once the sweet period is identified, grasp classification by the camera can be automatically conducted during this interval without purposed confirmation. In our recent publication (Wang et al., 2022), a similar sweet period (for sEMG) right before the hand grasps the target object was identified for hand grasp classification by sEMG. It will be interesting to explore the vision sweet period again during the reach-and-grasp process and utilize both of them for better prosthetic hand control.

In order to achieve the aforementioned analysis, we conducted an experiment to analyze the vision performance and find the sweet period with the best grasp type classification outcome. We first extracted object photographs from the original dataset to build a new dataset. Then, we fed a sequence of object images during the reach-and-grasp process to a deep learning model and output classified grasp types. The grasp classification accuracy and the ratio of the number of images containing objects to the total images are analyzed along the whole reach-and-grasp process to identify the vision sweet period. Finally, we integrated sEMG and vision classification outcomes to identify a better classification strategy. We hypothesize that the sweet period is at the beginning of the reaching phase when the target object has a higher probability of being visible to the participant, and the integration can provide higher accuracy of grasp classification.

## 2 Materials and methods

### 2.1 Dataset

**FIGURE 1:**
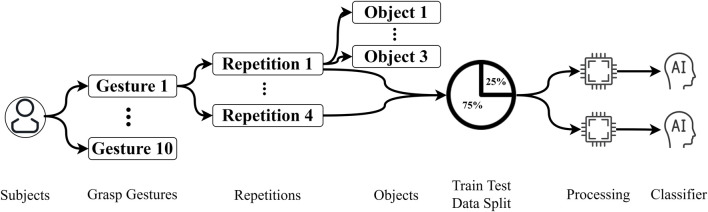
Data structure and processing steps.

The main dataset employed in this study is from Cognolato et al. (2020), which contains sEMG signals and a simultaneous first-person video. Ten grasp gestures were performed on 18 different objects in this data collection which was selected based on the grasp taxonomies (Cutkosky, 1989; Crawford et al., 2005; Sebelius et al., 2005; Feix et al., 2016) and grasp frequencies in activities of daily living (Bullock et al., 2013). As shown in Figure 1, the participant performed each gesture on three different objects, and on each object, the same grasp gesture were repeated four times. The list of gestures and objects is shown in Table 1. In addition to sEMG signals and videos, simultaneous eye-tracking data are also included in this dataset which we did not utilize in our research.

**TABLE 1:** Columns indicate the ID, the name of the grasp gestures, and the name of the object involved in the grasping (Cognolato et al., 2020).

**ID**	**Grasp gesture**	**Object**
1	Medium wrap	Bottle
		Can
		Door handle
		Mug
2	Lateral	Key
		Pencil case
		Plate
3	Parallel extension	Book
		Drawer
		Bottle
4	Tripod grasp	Mug
		Drawer
		Ball
5	Power sphere	Bulb
		Key
		Jar
6	Precision disk	Bulb
		Ball
		clothespin
7	Prismatic pinch	Key
		Can
		Remote
8	Index finger extension	Knife
		Fork
		Screwdriver
9	Adducted thumb	Remote
		Wrench
		Knife
10	Prismatic four finger	Fork
		Wrench

Each subject performed 10 grasp gestures in the original video, and each grasp was acting on three different objects. On each object, the same grasp gesture was repeated four times. There were 30 subjects in the database. Therefore, there were 3,600 grasp trials in total (120 trials x 30 subjects) in this dataset. Therefore, for each subject, there were 120 (10 × 4 × 3) grasp trials in total. Each grasp trial lasted approximately 4.5–5 s (Cognolato et al., 2020). To keep all the trials to the same length, we removed the frames after 4.48 s. Please be noted that the video was recorded with a frame rate of 25 Hz (one frame per 0.04 s), and there were 112 frames in each trial of the video.

### 2.2 Object detection using a RetinaNet model

We used RetinaNet to detect objects from the frames of the video. RetinaNet is a one-stage convolutional neural network model that has been widely used for object detection, which utilizes a focal loss function to address class imbalance during training (Lin et al., 2017). Considering the high volume of the dataset and the heavy labeling work, in this study, we chose the RetinaNet model pre-trained by the COCO dataset to reduce the required size of the training dataset. The COCO dataset included photographs of 91 object types that would be easily recognizable by a 4-year-old, and it contained a total of 2.5 million labeled instances in 328k images (Lin et al., 2014).

Because the object types in the dataset we used were not fully covered by the COCO dataset, fine-tuning is required to make it fit our object types. We built a dataset by collecting and labeling 1,186 photographs from the videos of the 30 subjects. There are 3–6 objects in each photograph, and each object showed approximately 200 times in these 1,186 photographs. Here, 80% of this dataset was used for training, and 20% was used for validation. Then, we created a new output layer to replace the previous output layer in the pre-trained model and trained it using the training and testing data mentioned earlier (these training and testing data have no overlap with the data for classification training and testing in the following work). Therefore, the final model we obtained can be used to detect the object in each frame.

There could be multiple objects appearing in a frame of the first-person video, but we only needed to detect the target object that the participant was trying to grasp using the fine-tuned RetinaNet model (as described in section 2.1), where the target object was cropped using a bounding box. If the target object was shown in the frame, we regarded this frame valid. If the target object was not shown in the frame or entirely blocked by the hands, we regarded it invalid. Setting up the valid frame where the target object is shown within the frame was important for our further analysis because the object and gesture recognition model we applied for only worked under the condition where one object is presented in the frame. Objects could be detected by the RetinaNet model from approximately 90% of the frames. We reviewed and manually detected the object on the rest of the frames.

### 2.3 Grasp classification using the dual-channel CNN model

Zhang et al. (2022) proposed a dual-channel convolutional neural network (DcnnGrasp), in which object category information was adopted to improve the accuracy of grasp pattern recognition. To maximize the collaborative learning of object category classification and grasp pattern recognition, they further proposed a loss function called Joint Cross-Entropy with Adaptive Regularizer (JCEAR) derived from maximizing a posterior. The regularization coefficient and trainable parameters in the loss function JCEAR and DcnnGrasp were updated by a developed training strategy. From the experiments given in their study, it can be seen that, compared with SOTA methods, DcnnGrasp achieved the best accuracy in most cases (Zhang et al., 2022).

In this study, we trained the DcnnGrasp model by using the object photographs from the first three repetitions and tested by using the remaining one repetition. When applying this model to testing data, the input photographs in the same grasp trial were fed to the model chronologically, in which way we could obtain the outcome also in a time sequence.

### 2.4 Grasp phases

**FIGURE 2:**
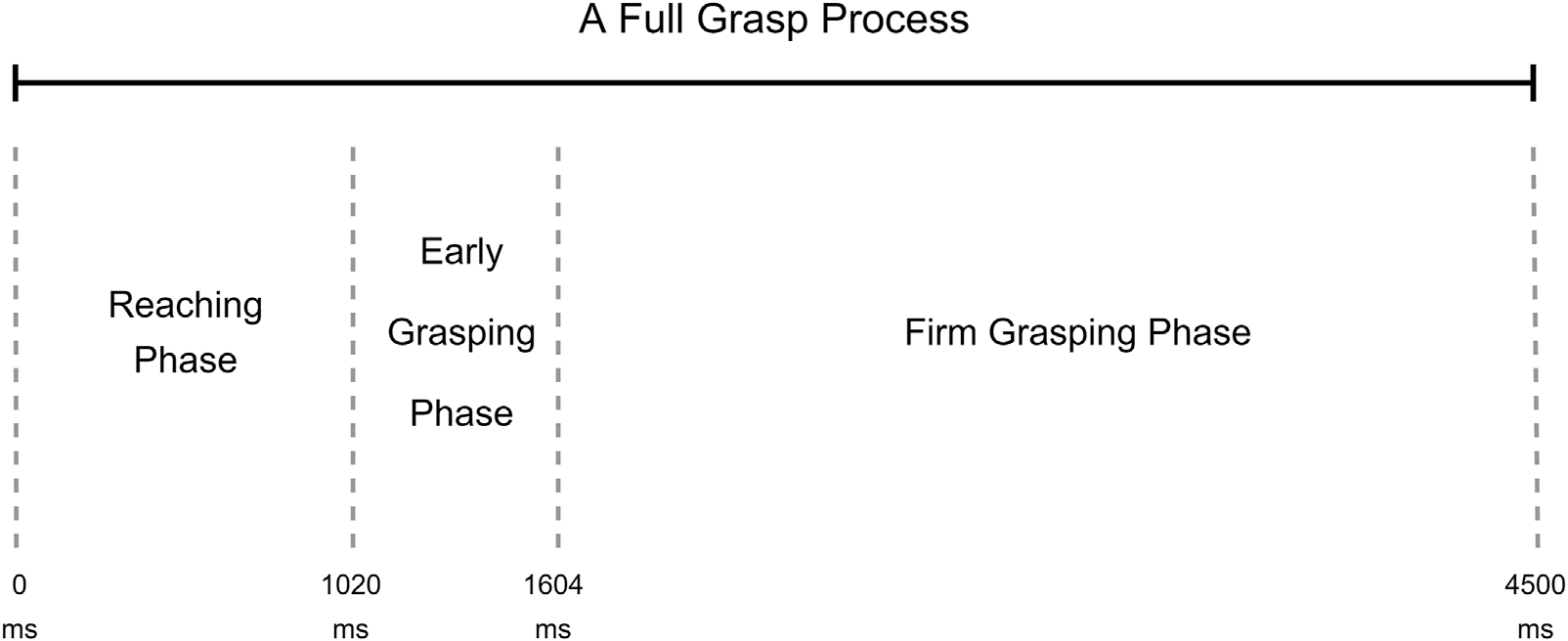
Example of grasp phases during a full grasp trial (Wang et al., 2022).

To better understand how the grasp classification models behave during the grasping process, we divided the entire grasping into three phases: the reaching phase, the early grasping phase, and the firm grasping phase. According to the research of Mason et al. (2002) and Supuk et al. (2011), the reaching phase starts with hands lifting and ends at touching the object, the early grasping phase starts with the object touching and ends by firmly holding the object, in the firm grasping phase, the force from hand remains steady to the end of grasp. The details of these three phases were discussed in our previous research (Wang et al., 2022). According to the segmentation for this dataset in our previous research, the reaching phase was from 0 to 1,020 ms, the early grasping phase was from 1,020 ms to 1,604 ms, and the firm grasping phase was from 1,604 ms to 4,480 ms. A phase example can be found in Figure 2.

## 3 Experiments and results

**FIGURE 3:**
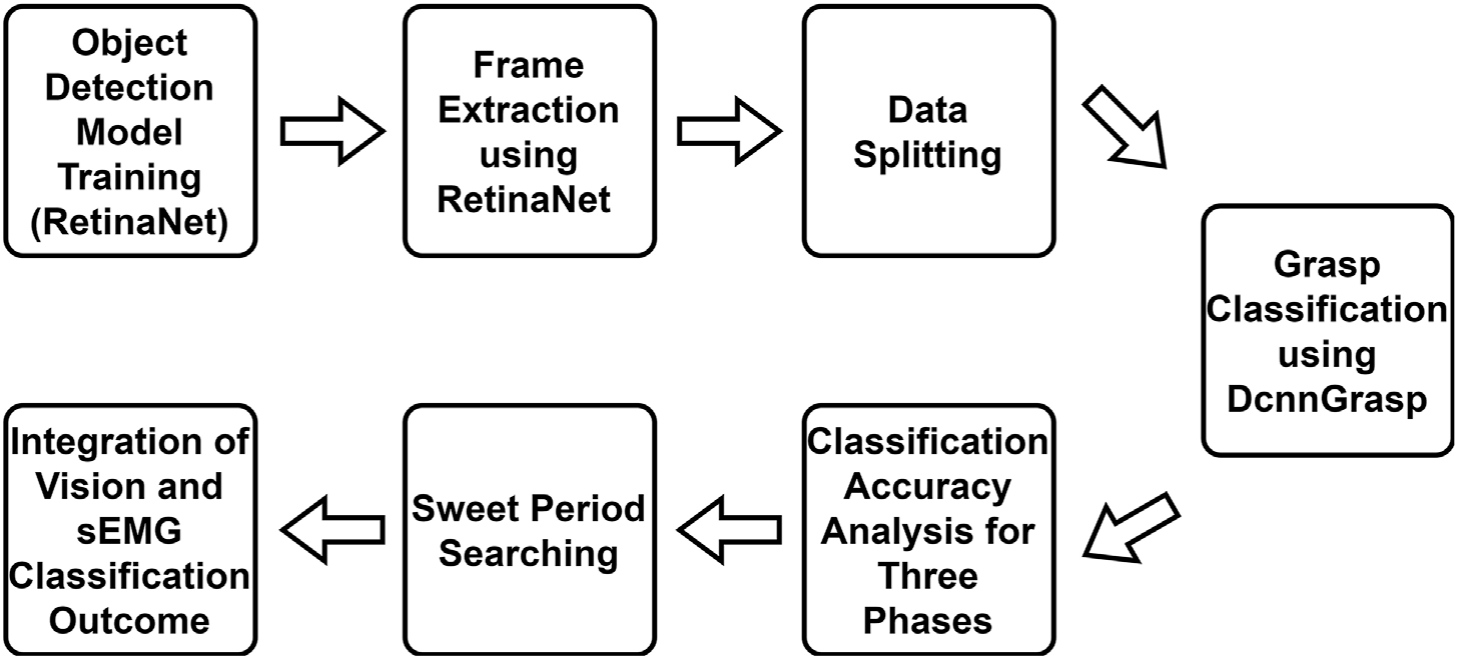
Workflow of this study. Object detection model training, frame extraction, and DcnnGrasp model are discussed in the Materials and methods section. The remaining four parts are discussed in the Experiments and results section.

The main workflow of this study is shown in Figure 3. In addition to the RetinaNet model, DcnnGrasp model, and frame extraction discussed in the previous section, another four steps will be introduced in this section.

### 3.1 Data splitting

As is mentioned in the previous section, there were four repetitions in each gesture performance process, allowing us to split the data into training and testing sets. We used three repetitions for training and one repetition for testing with leave-one-repetition-out cross-validation, in which each of the four repetitions has been regarded as the testing data once to ensure the reliability of the experiment. The data organization and processing steps can be easily understood from Figure 1.

### 3.2 Sweet period analysis

**FIGURE 4:**
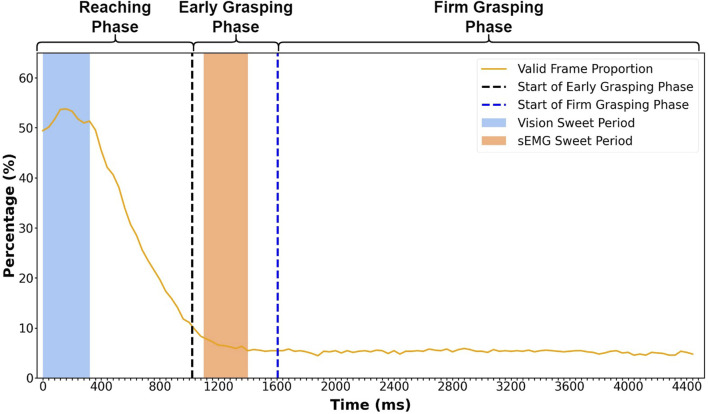
Valid frame proportion at each time point during the entire grasping process. The percentage represents the average proportion of valid frames in 900 trials from 30 subjects. The *x*-axis contains 112 points representing 112 frames in a grasp trial (40 ms for each frame, 4,480 ms in total). The vision sweet period starts from 0 ms and ends at 320 ms. The sEMG sweet period starts at 1,100 ms and ends at 1,400 ms. The vertical dashed lines are averaged starting times of the early grasping and firm grasping phases, which locate at 1,020 ms and 1,604 ms, respectively. The result is calculated with leave-one-repetition-out cross-validation.

After extracting the frames from the first-person videos, we calculated the proportion of valid frames and drew Figure 4 to illustrate the changes during the entire grasp process. There are 900 trials in testing data (30 subjects x 10 grasp gestures x 1 test repetition x 3 objects), which means that there are 900 frames at each time point. The percentage in Figure 4 represents the proportion of valid frames among these 900 frames at each time point. The result is calculated with leave-one-repetition-out cross-validation.

We can see that the valid frame proportion increases at the beginning of the reaching phase (Figure 4), reaching the peak at 160 ms. The valid frame starts to decrease until the late early grasping phase, keeping a stable low level during the entire firm grasping phase. The high percentage of the valid frame at the early reaching phase allowed us to define the location of the sweet period.

In searching for the sweet period, we defined several windows with different lengths and calculated the average percentage of valid frames in these windows. Since we wanted the sweet period to locate as early as possible and the percentage is high enough at the start of the reaching phase (0 ms in Figure 4), we made all the windows start from 0 ms and end at different times. After calculation, the window with the second highest average accuracy was chosen as the sweet period shown in the blue zone, which was from 0 to 320 ms (Figure 4). The window with the highest average accuracy (from 0 to 160 ms) was dropped because it only contained four frames which were not enough to make it reliable.

In our previous research (Wang et al., 2022), we achieved the best grasp classification outcome using the sEMG sweet period between 1,100 ms and 1,400 ms in the early grasping phase (pink zone in Figure 3). The sEMG sweet period was 800 ms behind the vision sweet period. The time gap between these two sweet periods makes it possible for us to integrate the classification outcome by vision and sEMG in real-life situation. Although the definition of a sweet period for sEMG and vision was the same, the methods to determine the sweet period were different. The sEMG sweet period was identified by analyzing classification accuracy, while the vision sweet period was found by analyzing the percentage of a valid frame.

### 3.3 Comparison of grasp classification performance

**FIGURE 5:**
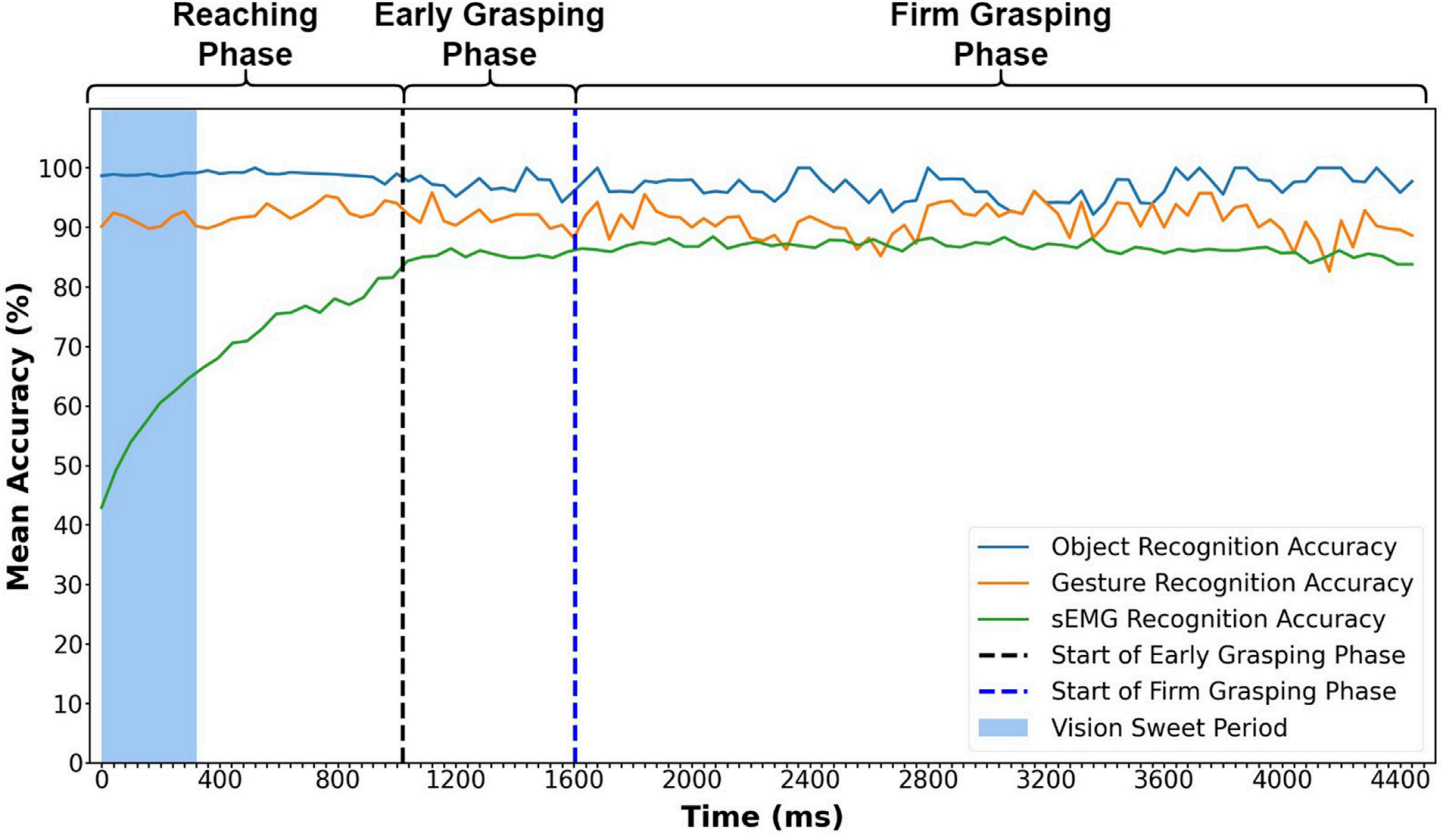
Mean accuracy for sEMG, object, and gesture classification at each time point during the entire grasping process. The object and gesture recognition results are from the trained dual-channel CNN model with leave-one-repetition-out cross-validation among valid frames. The mean accuracy represents the average accuracy of 900 trials from 30 subjects. The result is also calculated with leave-one-repetition-out cross-validation.

Figure 5 shows mean accuracy rates for grasp classification by three different methods (object recognition, gesture recognition, and sEMG) over the entire grasping phases. The value at each time point is averaged across all 900 trials from 30 participants. From Figure 5, we can see that object and gesture recognition accuracy remain relatively stable, fluctuating slightly between 97 and 91%, and are both higher than sEMG recognition accuracy in most circumstances. The object classification yields a higher accuracy than the gesture classification during the entire grasp process and only reverses once at 3,200 ms. The accuracy determined by the gesture classification only goes below the classification by sEMG on three occasions during the firm grasping phase. Overall, the gesture classification accuracy is much higher than sEMG recognition in most time.

**FIGURE 6:**
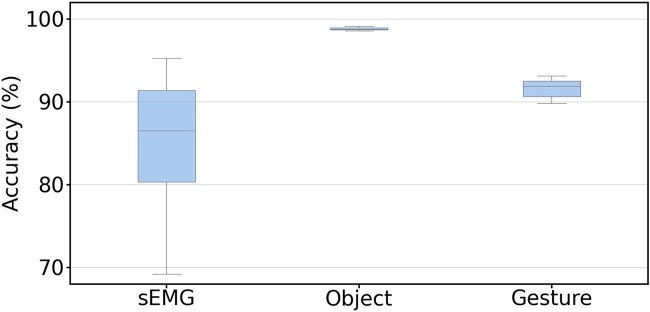
Mean classification accuracy of the object and gesture during the sweet period among 30 subjects. The sEMG result is from the best strategy we achieved in our last research which also utilized the sweet period for sEMG signals. The values for sEMG, and object and gesture classification accuracies are 85.50, 98.81, and 91.59%, respectively. The result is calculated with leave-one-repetition-out cross-validation.

Our last comparison of grasp classification over three methods focused on the sweet periods. Specifically, we calculated the mean accuracy of object and gesture recognition using data collected in their sweet periods and compared it to classification by sEMG from the best strategy we have achieved in the previous report (Wang et al., 2022). The results are shown in Figure 6, in which the object and gesture classification accuracy was calculated from valid frames in the sweet period. The mean accuracy reaches 98.81 and 91.59% for the object and gesture classification, respectively. Both were higher than the classification by sEMG (85.50%).

### 3.4 Integration of classification outcomes by vision and sEMG

Satisfied grasp classification outcomes from visual data encouraged us to integrate the gesture data from the vision sweet period with the sEMG data from the sEMG sweet period (Wang et al., 2022). The simplest and most effective method to integrate these two outcomes was comparing the plurality vote probabilities during their respective sweet period. For the visual part, the probability was calculated from the plurality vote results of the gesture recognition outcome only for the valid frames during the sweet periods. For the sEMG part, the probability was also calculated from the plurality vote results during the sweet period, but each time point in the sweet period was valid. Both probabilities illustrated the confidence of the grasp recognition results classified from sweet periods. We calculated and compared the probabilities for each grasp trial in the testing dataset and chose the outcome with the higher probability as the final classification result. After calculating the mean accuracy of all the 900 grasp trials in the testing dataset with leave-one-repetition-out cross-validation, we obtained the results, as shown in Table 2. After the integration, the grasp classification accuracy was increased from 85.50 to 90.06%, as shown in Table 2; however, the result was 1.5% lower than the visual gesture classification.

**TABLE 2:** Gesture classification comparison. The result is calculated during the sweet period among 900 grasp trials from 30 subjects with leave-one-repetition-out cross-validation.

Gesture classification basis	Mean accuracy
sEMG	85.50%
Visual information	91.59%
Integration of sEMG and visual information	90.06%

The vision sweet period lasts for 320 ms, which means there are eight frames in this period. Because the valid frame proportion is approximately 53%, around half of the frames are invalid. For the circumstance that the number of valid frames is less than five and at least one frame failed the recognition, the probability would be equal to or less than 75%, in which sEMG would dominate this result because the probabilities of sEMG are stable and higher than 75% in most circumstances, according to our previous research (Wang et al., 2022). In the circumstance that no frame failed the recognition, the vision with 91.59% probability would dominate the result.

## 4 Discussion

Our hypothesis is supported by the results that there is a sweet period at the beginning of the reach phase where visual information can be used to classify grasp gestures by computer vision. Moving one step forward, visual information can be integrated with sEMG to further improve the performance of robotic hand implementation in real-life applications. This is important as the visual input can be used to improve the accuracy in predicting grasp patterns compared to only using sEMG signals. Furthermore, the sweet period of visual data is in the early reaching phase, which enables early grasp classification without a pause in the grasping action. This would make the prosthetic hand control more natural.

We found that the valid frame proportion reaches the peak at the start of the reaching phase, as shown in Figure 4. This is because, before the grasp manipulation, the subject would look at the object before executing the grasp action, making it possible for us to identify hand movement using the visual signal at this period. After reaching the peak, the proportion quickly decreases to approximately 10% at the end of the reaching phase. This is because, once the hand touches the object, some object starts to be fully covered by the hand, which blocks the object from showing in the subjects’ vision. Therefore, the RetinaNet model cannot detect the objects, and the classifier cannot process the recognition. Since the hand starts to touch the object during the reaching phase, the most dramatic proportion decline occurs in this period. During the firm grasping phase, hand occlusion happened frequently; only a few objects with considerable volume can be recognized, thus making the proportion retain a low level of less than 10%. Although the proportion is the highest during the sweet period, it is only 53%. This is because the value for each time point is calculated across the 900 grasp trials (from 30 subjects), in which the objects are not shown in the subjects’ vision at the current time point, or the objects are blocked by hand, making this frame invalid at this time point. For this proportion level, we can find that it is impossible to implement recognition at a particular time point for different subjects and objects. However, it is feasible to expand the time point to a period to implement the recognition. In this research, we call this period the sweet period and find it located between 0 and 320 ms. In this period, the probability of obtaining the valid frames is the highest compared to any other period, which means that this period can provide a stable input to the classifier when performing a grasp action naturally.

From Figure 5, we can find that the visual recognition accuracy is much more stable than sEMG recognition accuracy in the reaching phase. This is because sEMG signals change very much with muscle contraction, but visual information changes are rare, with only some minor changes of the visual angle. Therefore, as long as the visual information input is enough, the classification outcome would be stable.

As we mentioned in the previous section, there is approximately 800 ms time difference between the sweet periods of sEMG and visual information. Therefore, in real-life applications, we can obtain the vision recognition result before processing classification by sEMG and integrate these two classification outcomes without causing a delay in myoprosthetic hand control the real-life applications. After the integration, the accuracy increases from 85.50 to 90.06%, from which we can find that, as a second role, visual information can effectively increase the overall gesture classification accuracy, thus increasing the performance of the sEMG prosthetic hand.

This study has some limitations. First, only one target object was chosen by the RetinaNet model to simplify the experiment. In the future, we could use gaze tracking technology to identify the target object in prosthetic hand control. Second, we only used one head-mounted camera for capturing the visual information. With the fast developing ubiquitous computing technology, multiple cameras will be available on the prosthetic hand or the ambient environment. Thus, we will collect more data including real-time tasks, to further improve and validate the system, such as using multiple channels’ visual information to enhance prosthesis control and exploring the corresponding sweet period/periods. Last, considering the fact that this study focuses on finding the best duration for vision grasp classification and its integration strategies with sEMG, we have not validated our results on the unseen objects in our study. The recognition accuracy might be affected when dealing with unseen objects.

## 5 Conclusion

In order to increase the performance of myoprosthetic hand control in real-life situation by integrating visual information to sEMG, we investigated the object and gesture recognition performance during the entire natural grasp process to identify the sweet period for grasp classification. We found that the sweet period is between 0 s and 320 ms from the start of the hand grasping, which happens in the reaching phase. Furthermore, we found that using visual information can yield higher classification accuracy. When integrating gesture recognition and classification by sEMG, we achieved an improved performance of myoprosthetic hand control.

## Data Availability

Publicly available datasets were analyzed in this study. This data can be found here: Harvard Dataverse, https://dataverse.harvard.edu/dataverse/meganepro.
